# Identifying person misfit using the person backward stepwise reliability curve (PBRC)

**DOI:** 10.3389/fpsyg.2023.1273582

**Published:** 2023-10-12

**Authors:** Georgios Sideridis, Fathima Jaffari

**Affiliations:** ^1^Boston Children’s Hospital, Harvard Medical School, Boston, MA, United States; ^2^National and Kapodistrian University of Athens, Athens, Greece; ^3^Education and Training Evaluation Commission, Riyadh, Saudi Arabia

**Keywords:** person reliability, K-R 20, aberrant responding, person fit, visual analysis

## Abstract

The goal of the present study was to propose a visualization of aberrant response patterns based on the idea put forth by the Cronbach-Mesbach curve. First, an index of person reliability is developed using the K-R 20 formula followed by a backward stepwise procedure in which one person at a time is deleted from the model. Observations for which reliability is no longer monotonically increasing suggest that they are candidates for aberrant responding. Using data from the quantitative domain of a national aptitude test the proposed visualization technique was demonstrated. The external validity of the procedure was tested by contrasting the person fit reliability estimates with those derived from other indices of aberrant responding such as the Ht. Results indicated that individuals not covarying with other individuals concerning their response patterns and concordance to the measurement of a unified latent trait were identified by both the present procedure and Ht and U3 at a rate of 100%. By plotting those individuals using Person Response Curves (PRCs) results confirmed the lack of monotonicity in the relationship between item difficulty and person skill. Consequently, results confirm the usefulness of the present methodology as an index for identifying responders who manifest themselves with aberrant responses and who are not conducive to the measurement of the latent trait.

## 1. Introduction

When individuals take a test, several processes are operative that may affect the way of responding which may result in the provision of invalid results. This notion of behaving in aberrant and unexpected ways represents a serious threat to the validity of test results with significant implications for both the person and the instrument (Little and Moore, 2013; Ferro and Beaton, 2016) as test scores include construct-irrelevant variance (Messick, 1995). At the personal level, individuals may obtain results substantially higher (as in cheating-see Cizek, 1999) or lower (as in being inattentive and careless, Meade and Craig, 2012) with significant implications for placement, selection, academic and job opportunities, etc.

Types of aberrant response patterns may involve random guessing (Lord, 1964), withdrawal (Ward et al., 2017), carelessness (Rios et al., 2017), speeding (Wise and Kong, 2005), rapid guessing (Deribo et al., 2021), inattentiveness (McKay et al., 2018), the presence of acquiescence (Plieninger and Heck, 2018), faking (Paulhus, 1991), social desirability (Leite and Cooper, 2010), recall biases (Barry, 1996), random responding (Cook et al., 2016), non-responding (Groves, 2006), ineffective strategy use (e.g., skipping items), the engagement of response sets (Müller et al., 2015), extreme responding (Meisenberg and Williams, 2008), response drifting (Drasgow and Parsons, 1983), insufficient effort (Hong et al., 2019), insufficient responding (Bowling et al., 2016), etc. Regardless of whether such behaviors are intentional or not, they have a major impact on the reliability and validity of the obtained scores. Thus, it is important to have tools to identify aberrant responses so that processes may be put in place to address the validity of test scores as they reflect the person or the instrument in total and likely represent a major threat to validity (van Laar and Braeken, 2022).

### 1.1. Reliability in measurement and aberrant responding

Ultimately, the quality of measurement is expressed by the ability of an instrument to provide measurements that are accurate, precise, and repeatable. This concept of reliability of measurement is most often discussed and estimated using information derived from a sample on a scale’s components, such as the items. One of the proponents of internal consistency reliability was Cronbach (1951) who also proposed the alpha coefficient as a reflection of the strength of the relationships between a set of items and the measured construct, assuming unidimensionality. Alpha is expressed using the following formula:


(1) $$\alpha =\frac{k}{k-1\;}\left(1-\frac{\sum \sigma_{i}}{\sigma_{y_{i}}^{2}}\;\right)$$

With *K* being the number of the items in the scale; and $\sigma_{i}\;\mathrm{and}\;\sigma_{y_{i}}^{2}$ the item’s variances and total variance, respectively. As a means to improve the internal consistency of a measure that does not reach acceptable standards, an item analysis methodology termed “reliability if item deleted” has been proposed so that one item at a time is excluded and alpha is re-expressed with the remaining items. The value of alpha is then evaluated with and without the removed item and decisions regarding internal consistency and unidimensionality are based on those estimates.

Mesbah (2010) put forth a graphical method using the logic of “alpha if item deleted” for evaluating the unidimensionality of a set of items. This stepwise method engages the “Backward Reliability Curve – *BRC*” with alpha being graphed after each successive step. Initially, the value of alpha is calculated using all items of a latent variable. After that, one item would be removed at a time with the value of alpha being re-estimated with the remaining items. The selection of the item in a stepwise fashion is based on the one that maximizes alpha if the item is deleted. Thus, the stepwise method concludes when only two items remain. Based on Classical Test Theory (CTT) and the Spearman–Brown formula, adding more items to the scale increases its reliability, thus a monotonically increasing BRC is expected when all items contribute to the formation of a unidimensional latent variable.

The present study extends the idea of the BRC at the person level by graphing a scale’s reliability using a person-deleted stepwise procedure and plotting the reliability of a measure by examining how each person contributes to the measurement of a reliable unidimensional structure. In other words, the goal of the present graphical person-deleted alpha is to identify, and subsequently discard, individuals who behave in ways that the reliability of a measure is compromised. This procedure provides information about the sensitivity of the measure to individual responses by identifying individuals with aberrant response patterns that deviate markedly from the model’s expectations (see Meijer, 1994). Thus, the original graphical method can be applied at the person level with the difference being that instead of removing \ adding one item at a time, we remove \ add one person at a time. Any decrease in the value of the reliability of the measure and the monotonic relationship expected by the BRC would be indicative of a person that is not constructive for measurement purposes or otherwise, that his/her response pattern reflects aberrant responding such as inattention or carelessness (Kam and Chan, 2018). To validate the proposed methodology, we employed a person-fit analysis with a known index that evaluates aberrant responding patterns. A substantial overlap in the selection of individuals who behave in unexpected ways following the Guttman pattern using the person BRC, and person fit statistics would provide evidence for the validity of the proposed methodology. Furthermore, by employing Person Response Curves (PRCs) the presence of aberrant responding will be evident in individuals whose curve does not conform to the descending trend as item difficulty increases. Thus, the goal of the present study was to introduce the Person Backward Reliability Curve (PBRC) and examine its criterion-related validity of selected misbehaving individuals in relation to the Ht index (Meijer and Sijtsma, 2001) and using Person Response Curves (PRCs).

## 2. Method

### 2.1. Participants and measure

Participants were *n* = 82 students who were part of a pilot study to evaluate general aptitude using the General Ability Test (GAT) which is a national criterion for university admission in Saudi Arabia. The quantitative domain utilized here was comprised of 44 items using a dichotomous scaling system. The quantitative domain assesses arithmetic, number sequence, analysis, logic, inductive reasoning, spatial ability relations, and visualization and is reflective of a single general dimension. In the present study we tested for the unidimensiionality of the measure by choosing among competing models using modern psychometrics.

### 2.2. Data analyzes

Three types of person-based analyzes for investigating aberrant response patterns were engaged, (a) the person backward reliability curve (PBRC), (b) the visual analysis of Person Response Curves (PRCs), and (c) the analysis of response vectors using person fit indices such as the Ht (Meijer and Sijtsma, 2001) and U3 (Van der Flier, 1982). The level of significance was set to 5% for a two-tailed test. In the presence of a family of tests (e.g., Table 1), we corrected for family-wise error using the Benjamini Hochberge corrective procedure. We opted against the popular Bonferroni procedure due to its conservatism and the fact that it does not adequately control for the false discovery rate (Holm, 1979; Nakagawa, 2004).

**Table 1:** Item fit statistics for quantitative domain, discrimination, and item difficulties.

Item No.	*X* ^2^	*d.f.*	Value of *p*	p-BH	a	*s.e.*	*b*	*s.e.*
6	6.520	1	0.011	0.294	2.440	1.050	−1.810	0.400
10	25.540	13	0.020	0.294	0.790	0.290	0.700	0.400
19	13.560	6	0.035	0.294	1.120	0.550	−2.230	0.840
8	6.230	2	0.044	0.294	2.120	0.890	−1.710	0.400
39	21.730	13	0.060	0.294	1.400	0.420	−0.330	0.210
20	17.610	10	0.062	0.294	1.570	0.450	−0.320	0.200
14	11.870	6	0.065	0.294	3.010	0.940	−0.670	0.160
5	23.610	15	0.072	0.294	0.560	0.260	−0.100	0.420
4	15.540	9	0.077	0.294	1.400	0.390	0.500	0.260
32	16.160	10	0.095	0.294	0.960	0.320	0.600	0.340
37	17.270	11	0.100	0.294	1.160	0.360	0.020	0.250
23	15.880	10	0.103	0.294	1.580	0.440	0.220	0.220
13	14.450	9	0.107	0.294	2.070	0.610	−0.570	0.180
24	21.970	15	0.108	0.294	0.590	0.270	−0.190	0.410
21	14.700	10	0.143	0.362	1.190	0.350	0.450	0.280
25	10.420	7	0.165	0.374	1.360	0.420	1.450	0.410
17	17.530	13	0.176	0.374	0.580	0.300	−1.420	0.730
2	16.090	12	0.187	0.374	1.160	0.350	0.400	0.280
27	14.890	11	0.187	0.374	0.990	0.380	−1.220	0.410
36	16.230	13	0.236	0.449	0.300	0.270	−3.190	2.870
16	12.290	10	0.266	0.461	0.970	0.380	−1.310	0.460
38	8.790	7	0.270	0.461	1.770	0.590	−1.140	0.260
12	16.570	14	0.279	0.461	0.580	0.270	1.110	0.650
31	12.530	11	0.327	0.510	1.900	0.520	−0.060	0.190
30	5.720	5	0.336	0.510	3.230	1.040	−0.660	0.150
29	12.200	11	0.350	0.512	1.560	0.460	−0.450	0.200
15	12.240	12	0.428	0.599	0.810	0.340	−1.240	0.500
3	11.020	11	0.443	0.599	1.140	0.340	0.820	0.330
1	12.900	13	0.457	0.599	1.010	0.370	−1.070	0.380
33	15.470	16	0.492	0.611	0.620	0.260	0.590	0.470
34	11.160	12	0.516	0.611	0.900	0.310	0.620	0.360
28	13.920	15	0.533	0.611	0.660	0.290	−0.750	0.440
7	14.680	16	0.550	0.611	0.600	0.270	−0.540	0.440
26	14.510	16	0.562	0.611	0.400	0.270	−1.700	1.180
22	8.690	10	0.563	0.611	1.860	0.520	−0.220	0.190
11	–	–	–	–	7.190	6.110	−1.440	0.200
35	7.730	10	0.656	0.692	1.320	0.420	−0.640	0.240
18	8.780	14	0.846	0.868	0.920	0.320	−0.400	0.290
9	3.800	9	0.924	0.924	2.250	0.620	0.120	0.190

p-BH are *p*-values corrected using the Benjamini-Hochberg correction; a, discrimination parameter; b, item difficulty; c.s.e.m, conditional standard error of measurement.

#### 2.2.1. Backward reliability curve (BRC) and the person variant (PBRC)

The analysis based on the backward reliability curve originates from the work of Mesbah (2010) who attempted to graphically describe unidimensionality. He furthermore stated that a combination of items reflects a unidimensional construct if each item is related to the underlying latent dimension exclusively (Hamon and Mesbah, 2002). Furthermore, using Cronbach’s alpha he suggested that the internal consistency reliability of a measure tends to increase with an increase in the number of items. Graphically speaking he proposed the Backward Reliability Curve (BRC) that is being estimated in multiple steps with the first step including all items. Then at each subsequent step, one variable is removed from the model so that the variable selected is the one that results in the maximum value of Cronbach’s alpha. Given that a monotonic relationship must exist between the number of items and alpha if an item is associated with a decrease in the curve, then that item is suspected that it does not contribute to the latent construct under evaluation. Under those lenses, items that are not associated with increases in the BRC, are candidates for exclusion.

In the present study, we propose two modifications to the BRC. First, by transposing items and columns, the BRC would be reflective of individuals who are constructive for measurement purposes, hence the term Personal Backward Reliability Curve (PBRC). Thus, individuals that lead to BRC decays are suspect and subject to removal. Second, we substituted Cronbach’s alpha with the Kuder–Richardson estimation, which is appropriate for binary data (see Supplementary material on modification of CMC package functions). Consequently, the PBRC can utilize individuals who are only reflecting an increasing curve, thus, representing a more reliable measurement.

#### 2.2.2. Ht and U3 person fit indices

The Ht coefficient, as presented by Meijer and Sijtsma (2001), is a measure used to quantify the extent to which data adhere to the Guttman model (Guttman, 1944 Meyer et al., 2013) for a single respondent in comparison to the other respondents within a given sample. The Ht coefficient is calculated by summing the covariances between the respondent’s responses and the responses of the other respondents in the sample in the form of a covariance ratio as shown below:


(2) $$H^{T}=\frac{\mathrm{cov}\left(x_{n},r_{\left(n\right)}\right)}{{\mathrm{cov}}_{\text{max}}\left(x_{n},r_{\left(n\right)}\right)}\text{,}$$

With xn being the response vector for person *n*, and *r*(*n*) being the response vector of total scores calculated from every participant in the sample except the xn person. Karabatsos (2003) suggested a cutoff value of <0.22 for Ht.

The maximum possible value of the Ht coefficient is 1, which indicates that the respondent’s responses perfectly conform to the Guttman scale. A lower value of the Ht coefficient indicates that the respondent’s responses are less consistent with the Guttman scale with values greater than 0.3 being suggestive of acceptable levels (Wongpakaran et al., 2019) or greater than 0.22 (Karabatsos, 2003). Simulation studies have shown that it has a high level of accuracy in detecting aberrant responses when applied to data with dichotomous response scales across different settings (Karabatsos, 2003 Dimitrov and Smith, 2006 Tendeiro and Meijer, 2014). Ht does not have a known theoretical distribution thus tests of inferential statistics cannot be conducted compared to other indices (e.g., lz*, Snijders, 2001 Magis et al., 2012) but given its efficacy in past research, it will be used as one of our two golden standards to determine the criterion validity of the proposed PBRC methodology.

The second person-fit index utilized, the U3 statistic, was developed by Van der Flier (1982) and was found to be the most accurate for the detection of random responding (Karabatsos, 2003) compared to all other tested indices (*n* = 36). Several studies confirmed the efficacy of U3 as an index of inattentive responding (e.g., Beck et al., 2019). The index reflects the ratio of the actual number of Guttman errors in a response pattern relative to the maximum number of errors using the log scale (Emons et al., 2005). It is being estimated as follows:


(3) $$U3=\frac{f\left(x_{n}^{*}\right)-f\left(x_{n}\right)}{f\left(x_{n}^{*}\right)-f\left(x_{n}^{\prime }\right)}$$

With $x_{n}^{*}$ being the Guttman vector with correct responses for the easiest items in s_n_, $x_{n}^{\prime }$ the reversed Guttman vector with correct responses for the $s_{n}$ hardest items, and $f\left(x_{n}\right)$ being the summation $\overset{I}{\underset{i=1}{\sum }}x_{ni}\log\left[p_{i}/\left(1-p_{i}\right)\right]$ . In the Mousavi et al. (2019) study, the U3 index outperformed the Ht index across most conditions. Karabatsos suggested a cutoff value of 0.25 for U3 but Mousavi et al. (2019) challenged this cutoff value that was based on the standard normal and instead favored the value of p method and/or bootstrapping. All person fit indices were analyzed using the Perfit package (Tendeiro et al., 2016) in the R environment (R Core Team, 2017).

#### 2.2.3. Analysis of person response curves (PRCs)

As an ancillary way of evaluating and validating a person’s misfit, we will plot a person’s proclivity to success using Person Response Curves (PRC). PRCs represent graphical means to evaluate the probability of a person’s success on items of increasing difficulty. Thus, for any given individual, the expectation is that the curve will show a descending relationship with item difficulty by the use of an S-shaped curve. The curve is expected to start high as a person is likely successful on the easy items and is expected to gradually descend as the likelihood of correct responding goes down. Irregular PRCs would suggest that individuals are less successful on items that are within their level of ability and more successful on items that are out of reach, representing unexpected patterns more likely linked to inattention and/or cheating.

## 3. Results

### 3.1. Item response model for quantitative scale

A 2PL Item Response model was fit to the data and model fit was evaluated using descriptive fit indices and the RMSEA as well as the omnibus chi-square test. Results indicated acceptable model fit as the chi-square test was non-significant [*χ*^2^ (702) = 751.598, *p* = 0.95]. Furthermore, the CFI and TLI were 0.936 and 0.932, respectively. Last, the RMSEA point estimate was 0.029 (RMSEA_95%CI_ = 0.000–0.046). When contrasting the 2PL model to the fixed discrimination parameters model (Rasch), results indicated the superior fit of the 2PL model. Specifically, the Bayesian Information Criterion (BIC) values were 3732.72 for the 2PL model and 4073.19 for the Rasch model, suggesting the superiority of the former. Thus, collectively all information pointed to a good model fit using the 2PL model supporting the unidimensionality of the latent quantitative skills construct. Table 1 displays item-based parameters and item fit for the instrument under study. Related to item misfit, all the corrected item-fit statistics based on the chi-square test suggested that items fit the premises of the Item Response Theory (IRT) model well and specifically the Guttman related pattern. Supplementary Figure S1 shows the Test Information Function (TIF) of the measure which peaked close to zero or slightly less than that and decays as it moves away further from mean theta, as expected with estimates deviating markedly from the mean and becoming less precise.

### 3.2. Person-based analyzes

#### 3.2.1. Person backward reliability curve (PBRC) and person response curves (PRCs)

**Figure 1:**
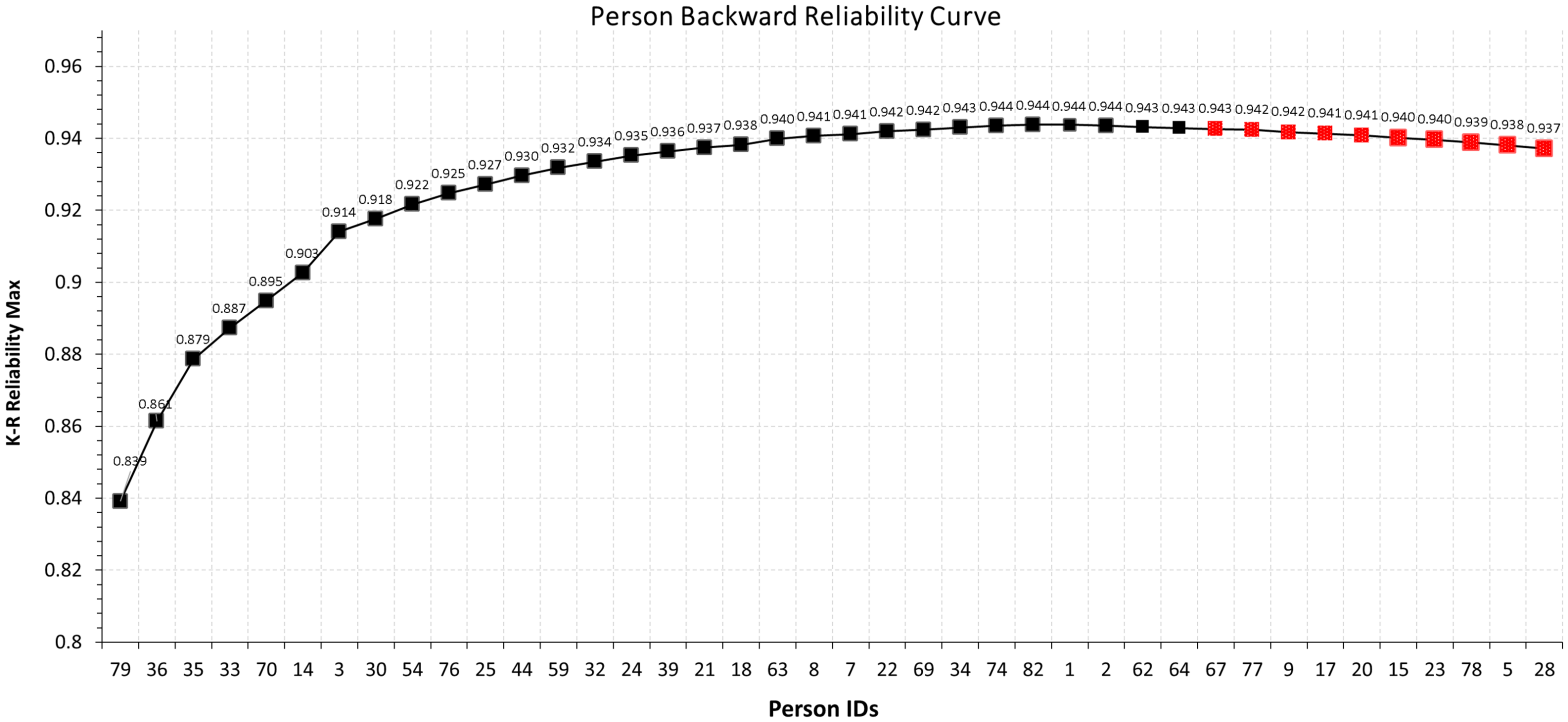
Modified Cronbach-Mesbach curve for the assessment of person reliabilities in relation to total person reliability using the K-R formula.

Figure 1 displays the proposed person backward reliability curve using fewer observations for illustration purposes. As shown in the figure, as participants are added to the measure so does internal consistency reliability which peaks at around 0.953 using the K-R formula. However, following that peak, the curve decays suggesting that the inclusion of specific individuals results in decrements in the model’s estimated reliability. These observations were persons with ids 28, 5, 78, 23, 15, 20, 17, 9, 77, and 67. Thus, by merely using graphical means, these participants contribute amounts of error that are linked to decay in the measurement of internal consistency reliability. In other words, these participants are not contributing valuable information to the measure’s reliability. Further analyzes of their response vectors highlight the possible causes for that misfit as highlighted by the PBRC.

**Figure 2:**
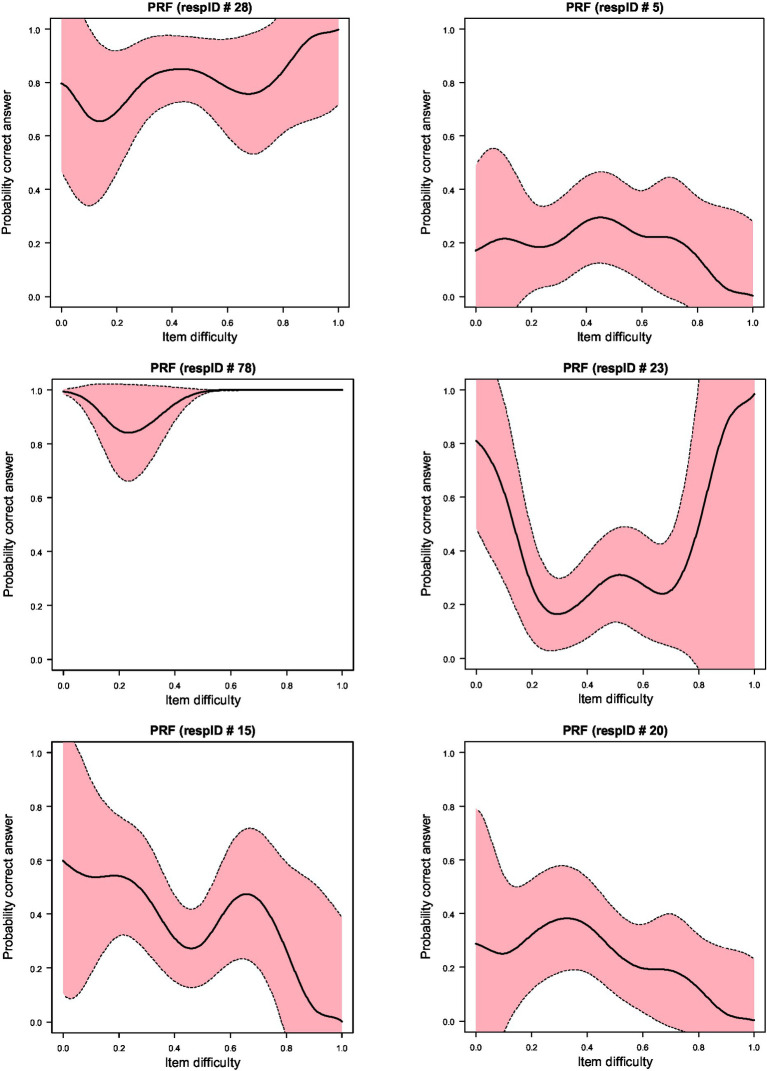

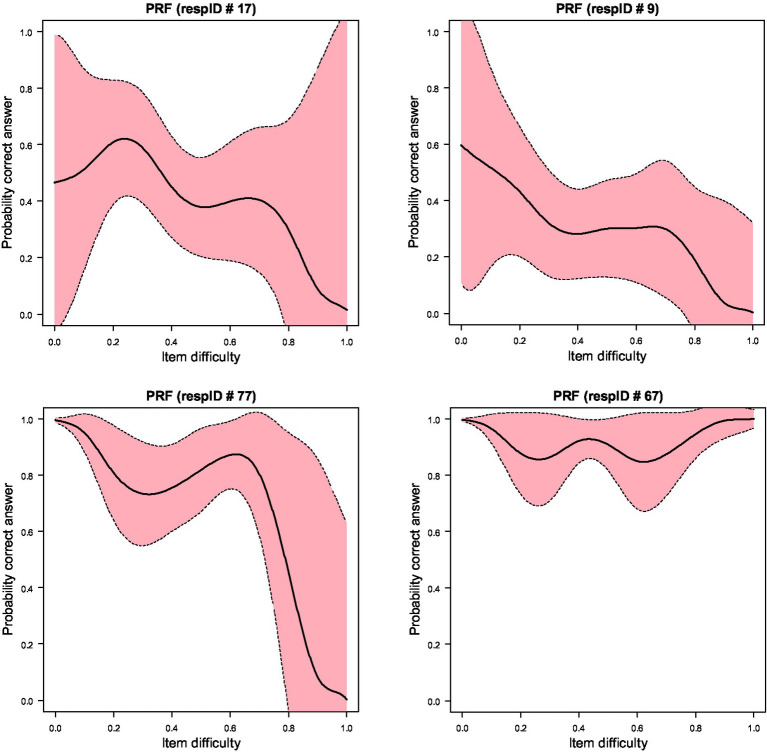
Person Response Functions (PRFs) for 10 of the most aberrant responders as identified using the sampling distribution of Ht using bootstrapping. Upper and lower confidence intervals (shaded area) are at 95%.

Figure 2 displays the Person Response Curves (PRCs) for the 10 responders who were associated with decrements in the PBRC in Figure 1. As shown in the figure no participant displayed a PRC that was S-shaped with decays associated with decreases in item difficulty levels. As an example, the PRC of the first individual, id 28, displays a wave-like pattern with actual increases in item difficulty being associated with increases in the probability of success, which, as a pattern of behavior is against any of the premises of item response models. Person 28 had a theta estimate of 0.81 (S.E. = 0.308), thus, representing an above-average ability individual, who, however, was more successful on items beyond her/his ability level likely reflecting cheating; furthermore, this participant was unsuccessful on items within her/his ability level, likely reflecting inattention.

#### 3.2.2. Person analysis of response vectors using Ht and U3

**Figure 3:**
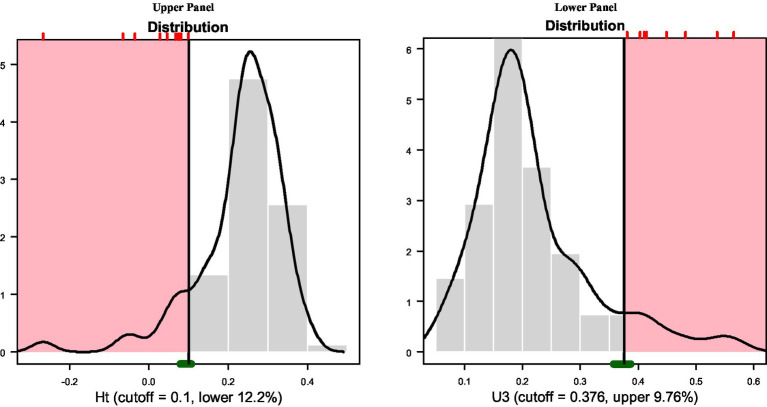
Distribution and cutoff value for Ht index.

As mentioned above, for the analysis of response vectors, the Ht coefficient was utilized given its efficacy in past research (Karabatsos, 2003) to identify aberrant responders specifically linked to lucky guessing and cheating. Misfitted participants were flagged using cutoff values of 0.10 based on bootstrapping to simulate the sampling distribution of the Ht index with the current sample at the predetermined level of significance of 5% (Tendeiro et al., 2016; Mousavi et al., 2019). Figure 3, upper panel, displays the bootstrap distribution of Ht and its cutoff level of 0.10 (upper panel). Interestingly, below the cutoff Ht estimate of 0.10, there were 10 participants, which were exactly those identified using the PBRC. The only difference was in the ordering of participants Ht flagging in order of aberrance participants 78, 28, 5, 67, 23, 15, 20, 17, 77, and last, participant 9.

Similar results were observed with the use of U3. Using a value of p of 5%, the U3 index flagged 8 participants utilizing a cutoff value of 0.376 based on the bootstrap distribution (see Figure 3, lower panel). These participants and in the order of aberrance were ids: 5, 9, 15, 17, 20, 23, 28, and 78. Thus, all 8 flagged participants using U3 were also identified by the Cronbach-Mesbach curve, again supporting the criterion validity of the proposed PBRC at a level of 80% as two participants were not flagged using the alpha level of 5%.

## 4. Discussion and concluding remarks

The goal of the present study was to propose a visualization of aberrant response patterns based on the idea put forth by the Cronbach-Mesbach curve. First, an index of person reliability is developed using the K-R 20 formula followed by a person backward stepwise procedure in which one person at a time is deleted from the model. The methodology was applied to the measurement of a quantitative skills latent trait using a sample of 82 participants. Results pointed to the usefulness of the PBRC in identifying aberrant response patterns by flagging 10 participants, who behaved in ways that deviated markedly from the Guttman pattern.

The most important finding of the present study was that the 10 participants flagged using the PBRC were the same 10 worst-fitted participants using the Ht index and were also among the 8 worse participants using the U3 index. Thus, the criterion-related validity of the PBRC was fully supported using Ht and also U3 at a level of 80%. Further, visual analyzes indicated that the PRCs of these participants reflected significant deviations between expected curves and those observed likely being reflective of the processes of lucky guessing (Foley, 2019) and carelessness or inattention (Meade and Craig, 2012; Maniaci and Rogge, 2014). Those participants were across the board of ability with theta values ranging between −1.71 and + 1.79, thus, the methodology was not sensitive to specific levels of person abilities, low or high. The present findings regarding the validity of the Ht and U3 indices corroborated with previous findings showing the superiority of these statistics compared to other alternatives (e.g., Karabatsos, 2003; St-Onge et al., 2011; Rupp, 2013; Tendeiro and Meijer, 2014; Beck et al., 2019; Mousavi et al., 2019; Wongpakaran et al., 2019).

The present study presents visual means to identify aberrant responding and is one of the available tools in data screening so that problematic responders are flagged and potentially removed. Novel ideas beyond person fit indicators involve simulation where response vectors are generated so that they mimic aberrant response patterns. Then these patterns can be evaluated for their presence with real data so that the detection of aberrant responders is achieved (Dupuis et al., 2018).

### 4.1. Limitations and future directions

The present study is limited for several reasons. First, the sample size was relatively small, and thus, results may have been idiosyncratic. Second, the selection of cutoff values of the person fit indices using bootstrapping represents only one among the different available methodologies (Mousavi et al., 2019). Third, the use of person fit indices is informative only *post hoc*; thus, they cannot inform individuals who may behave in aberrant ways before the study. Not only that but the estimation of person fit indices is based on the estimated item parameters that may also be biased by the presence of misfitting participants. Mousavi et al. (2019) proposed employing an iterative procedure, which may be both complex and cumbersome. Furthermore, as the sample sizes get large, the procedure may become cumbersome in terms of selecting criteria to flag aberrant responders and use criteria based on the level of significance and the expected number of outlying cases using the standard normal.

The currently proposed PBRC will need to be compared to additional aberrant responding indices in the future, such as lz*, and/or other indices that are intended to address particular cases of aberrant response and its underlying processes. The discriminant and predictive validity of the PBRC will need to be assessed in light of the effectiveness of other indicators of aberrant behavior. Future studies may also consider cutoff values and percentage of individuals classified as aberrant responders using both visual and statistical criteria. Additionally, a detailed evaluation of the PBRC’s capability and sensitivity to certain sorts of aberrant responses, such as inattention, carelessness, random responding, guessing, and cheating, is required. Researchers may examine the effectiveness of the PBRC in response to particular instances of aberrant behavior by methodically altering these parameters within experimental paradigms. This kind of study may provide crucial validity standards for assessing the PBRC’s performance and its capacity to precisely identify and evaluate aberrant responses in various circumstances, populations, and cultures (Van de Vijver and Tanzer, 2004). Researchers may create a framework that might result in the creation of new tools and practices to increase the accuracy and reliability of psychological assessments and educational evaluations by comprehending how PBRC matches with other indices of aberrant behavior (see Bereby-Meyer et al., 2002).

## Data availability statement

The raw data supporting the conclusions of this article will be made available by the authors, without undue reservation.

## Ethics statement

The studies involving humans were approved by Education and Training Evaluation Commission. The studies were conducted in accordance with the local legislation and institutional requirements. The participants provided their written informed consent to participate in this study.

## Author contributions

GS: Conceptualization, Formal analysis, Methodology, Writing – original draft, Writing – review & editing. FJ: Data curation, Formal analysis, Funding acquisition, Investigation, Methodology, Validation, Writing – review & editing.
