# Supplementary material for: Identifying person misfit using the person backward stepwise reliability curve (PBRC)
Source: Front Psychol. 2023 Oct 12;14:1273582. doi: 10.3389/fpsyg.2023.1273582 (PMC10602758; doi:10.3389/fpsyg.2023.1273582)
Supplement: Supplementary file 1 [file Data_Sheet_1.docx]

Supplementary Material

Identifying Person Misfit Using the Backward Stepwise Person Reliability Curve

Georgios Sideridis^1,2^*, Fathima Jaffari^3^

*** Correspondence:** Corresponding Author georgios.sideridis@childrens.harvard.edu

# Supplementary Tables

Modifications to the CMC package and specifically the “alpha.curve.r” function. Because the data in the present case were dichotomous, the Kuder-Richardson (K-R) formula was utilized. Thus, below the reader can find the original content of this function and the modified one:

Original alpha.cronbach function in the CMC package:

alpha.cronbach = function(x){

n = nrow(x) #n. of subject

k = ncol(x) #n. of items

var.cov.mat = cov(x) * (n-1)/n

num = sum(diag(var.cov.mat))

den = 2*sum(var.cov.mat[lower.tri(var.cov.mat)])+num

alpha = k / (k-1) * (1 - num/den)

return(alpha)

}

Modified alpha.cronbach function which returns the K-R coefficient:

alpha.cronbach = function(x, hit=1){

x<-na.omit(x)

n = nrow(x) #n. of subject

k = ncol(x) #n. of items

x <- data.frame(apply(x==hit, 2, as.numeric))

totalVar <- var(apply(x, 1, sum))

p <- colSums(x)/n

q <- 1-p

alpha <- (k/(k-1))*(1-sum(p*q)/totalVar)

return(alpha)

}

# Supplementary Figures

Supplementary Figure 1

*Test Information Function (TIF) of the Verbal Ability Scale.*
